# Incidence of a First Thrombo-Embolic Event in Patients With Systemic Lupus Erythematosus and Anti-phosphatidylserine/prothrombin Antibodies: A Prospective Study

**DOI:** 10.3389/fmed.2021.621590

**Published:** 2021-02-01

**Authors:** Savino Sciascia, Massimo Radin, Irene Cecchi, Elena Rubini, Silvia Grazietta Foddai, Alice Barinotti, Antonella Vaccarino, Daniela Rossi, Dario Roccatello

**Affiliations:** ^1^Department of Clinical and Biological Sciences, Center of Research of Immunopathology and Rare Diseases - Coordinating Center of Piemonte and Valle d'Aosta Network for Rare Diseases, Division of Nephrology and Dialysis, S. Giovanni Bosco Hospital, Turin, Italy; ^2^Division of Hematology, S. Giovanni Bosco Hospital, Turin, Italy

**Keywords:** antiphospholipid syndrome, antiphospholipid antibodies, anti-phosphatidylserine/prothrombin, aPS/PT, non-criteria aPL, thrombosis, systemic lupus erythematosus

## Abstract

**Objective:** This study aimed to prospectively investigate the incidence of first thromboembolic events (TEs) in a cohort of systemic lupus erythematosus (SLE) patients. The patients were positive for anti-phosphatidylserine/prothrombin (aPS/PT) antibodies and tested negative for anticardiolipin (aCL) and anti-β2–glycoprotein I (aβ2GPI) antibodies [regardless of their Lupus Anticoagulant (LA) status].

**Methods:** Inclusion criteria included: (a) SLE with no previous TEs; (b) no concomitant anti-thrombotic therapy; (c) isolated confirmed positive test for aPS/PT.

**Results:** From the total of 52 SLE patients (42, 80.8% women), 18 patients (34.6%) were found to be positive for aPS/PT (IgG/IgM). During a mean follow-up (3.9 ± 1.1 years), 3 TEs occurred (1.3%/year). The overall cumulative incidence of TEs was 5.8% after 2 years, and up to 16.7% when focusing on aPS/PT positive patients. All the TEs events (two cerebrovascular events and one thrombotic kidney microangiopathy) occurred in the aPS/PT positive group. When focusing on IgG aPS/PT, we found that patients who tested positive were at a significantly higher risk for TEs (crude HR 19.6, 95%; CI 1.1 to 357.6; *p* < 0.05) compared to patients with negative aPS/PT.

**Conclusion:** This study observed a rate of TEs of 1.3%/year, in aPS/PT positive only patients. Our prospective data suggest that aPS/PT might confer an increased risk for the development of TEs in SLE patients.

## Introduction

Multiple positivity in tests investigating the presence of antiphospholipid antibodies (aPL) [criteria aPL comprehend: lupus anti-coagulant (LA), anticardiolipin (aCL), and anti-β2–glycoprotein I (aβ2GPI) antibodies] are now widely recognized as being associated with a higher risk of developing thromboembolic events (TEs). The concomitant presence of all criteria aPL (triple positive patients) is associated with thrombosis and identifies high-risk patients in antiphospholipid syndrome (APS) setting (1). However, some individuals may show a clinical picture that strongly indicates APS even though they are persistently negative for criteria aPL tests. Current research examines testing for other aPL specificities to fill this diagnostic and therapeutic gap. When investigating these so-called “extra-criteria” aPL in a patient with clinical manifestations suggestive of APS, testing for anti-phosphatidylserine/prothrombin (aPS/PT) antibodies has been recommended as a further tool in guiding the management of these patients. It can be particularly relevant when there is an absence of criteria aPL or as a part of risk assessment approaches (2). This approach to testing has been analyzed by two systematic reviews (3, 4), which outline that aPS/PT antibodies might be considered a strong risk factor for TEs independently from sites and type of thrombosis. There is little data, available to provide prospective validation of the role the absence of other aPL tested by β2GPI-dependent assays.

This study prospectively investigates the incidence of first TE in a cohort of systemic lupus erythematosus (SLE) patients positive for aPS/PT antibodies who also tested negative for criteria solid assay (aCL and aβ2GPI antibodies), regardless of their LA status.

## Methods

### Inclusion Criteria

Since 2015, aPS/PT has formed part of routine testing in SLE patients as part of the autoantibody screening of consecutive patients attending the S. Giovanni Bosco Hospital (Turin, Italy). The patients included in this study were diagnosed with SLE according to the 1982 revised criteria (5), received prospective follow-up, and fulfilled the following criteria:

1. no previous TEs events;
2. no concomitant anti-coagulant nor anti-platelets therapy;
3. tested negative for criteria aPL solid assay aCL and aβ2GPI (confirmed at least twice, at least 12 weeks apart), regardless of their LA status.

All included patients were tested for aPS/PT, and both IgG and IgM, at study inclusion.

**Figure 1:**
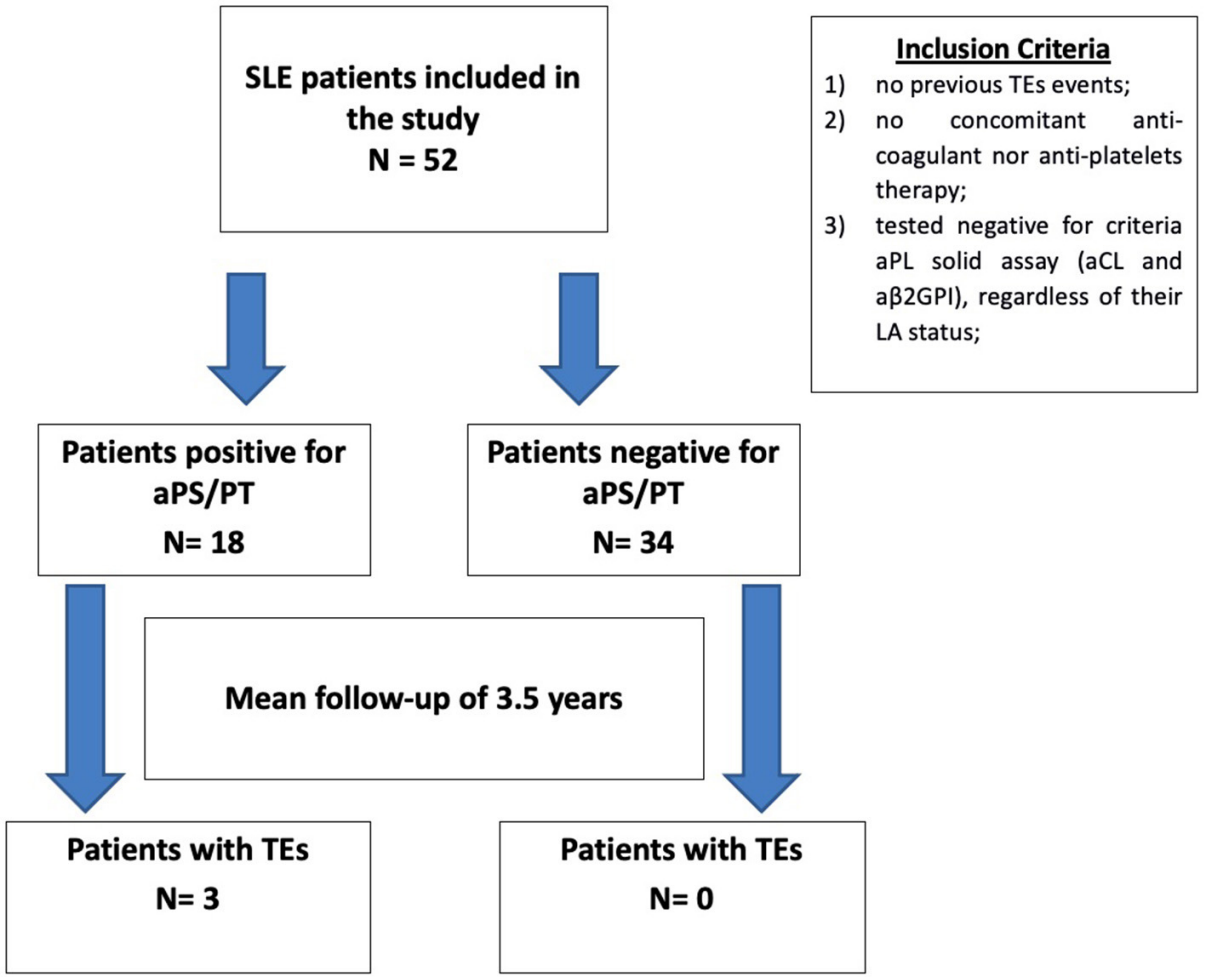
Patients disposition. Description of the patients selected for the study, according to the inclusion criteria. Patients included in the study were then separated according to their anti-phosphatidylserine/prothrombin status (positive/negative), and the number of thrombotic events was recorded retrospectively. aPS/PT, anti-phosphatidylserine/prothrombin; SLE, systemic lupus erythematosus; TE, thrombotic event; aPL, antiphospholipid antibodies; aCL, anticardiolipin; aβ2GPI, anti-β2–glycoprotein I; LA, lupus anti-coagulant.

Positive aPS/PT testing was defined as having at least two positive test results (IgG and/or IgM), at least 12 weeks apart. The disposition of patients is illustrated in Figure 1.

All subjects provided written consent according to the Declaration of Helsinki. This study was performed according to the local legislation of Rare Diseases in Piedmont (Northwest Italy) (protocol. n. 1577/UC/SAN 11.10.2005).

### Data Collection

Data on demographic, and laboratory and clinical features were prospectively collected every 6 months or at the time of any new clinical event for each patient. Patients with a previous history of TEs were excluded based on patient interviews and available hospital records.

Assessed arterial thrombotic risk factors were diabetes mellitus, arterial hypertension, hypercholesterolemia, obesity, smoking habit, and positive family medical history. Assessed venous risk factors were the following: ongoing hormonal replacement therapy, active pregnancy, malignancy, positive family medical history, and thrombophilia (including antithrombin, protein C, or protein S; factor V Leiden; prothrombin G20210A mutation; hyperhomocysteinemia, high factor VIII levels).

### Outcome Events

TEs had to be objectively diagnosed during the follow-up. TEs reports include type, site, SLE activity assessed by the Systemic Lupus Erythematosus Disease Activity Index 2000 (SLEDAI-2K) and ongoing medications at the time of event.

Venous thromboembolism (VTE) was assessed by compression ultrasonography or venography in case of deep vein thrombosis, and spiral tomography, ventilation-perfusion lung scan, or pulmonary angiography in case of pulmonary embolism. Intracerebral thrombosis was assessed by computed tomographic scanning, magnetic resonance imaging, or angiography; retinal thrombosis was evaluated by ophthalmologic examination. Peripheral- or mesenteric- artery thrombosis was documented by arteriography or at the surgery table. Small-vessel thrombosis was evaluated by appropriate imaging study or histopathology in the absence of inflammation in the vessel wall. Acute myocardial infarction was defined in the presence of a typical clinical presentation associated with typical electrocardiographic features and elevated cardiac enzymes (CK-MB or troponins I or T). Stoke/transient ischemic attack was defined according to standard definitions (transient ischemic attack was considered for analysis only if cerebral imaging confirmed cerebral ischemia).

### aPL Testing

Complete aPL profile at inclusion in the present study included: LA, aCL IgG/IgM, aβ2GPI IgG/IgM, aPS/PT IgG/IgM, and aβ2GPI Domain 1 (aβ2GPI-D1) IgG.

LA testing was performed according to international guidelines (6). Solid-phase aPL testing was executed with chemiluminescent immunoassay (INOVA Diagnostic) for aCL, aβ2GPI, and aβ2GPI-D1, while aPS/PT testing was performed using ELISA assay (INOVA Diagnostic). The cut-off values were determined by manufacturer recommendations. Cut-off values provided by the manufacturer were independently validated in a cohort of 100 healthy blood donors, and the used values were above the 99th percentile of the distribution. The Global APS Score (GAPSS) was calculated according to Sciascia et al. (7).

### Statistics

Descriptive statistics are reported as appropriate: categorical data are expressed as frequencies (percentage); continuous data are reported as mean ± *SD*. The Kaplan-Meier survival analysis was used to determine the cumulative incidence of TEs at follow-up. Student's *t*-test was used for normally distributed parameters and the non-parametric Mann–Whitney test for non-normally distributed parameters.

The Cox proportional hazards model was initially included in the statistical plan to detect possible predictors of TEs among the demographic factors. The initial model computed the following variables: age> 50 yrs, sex, active SLE assessed by SLEDAI-2K >6, and any additional thrombotic risk factor (smoking, arterial hypertension, hyperlipidemia, diabetes, immobilization). However, taking into account the rate of observed thrombosis, as the number of primary events per variable can affect the estimation of the subdistribution hazard competing risks model, we decided to keep this analysis as exploratory. A Log-rank test was performed, comparing thrombotic events during the follow-up according to the aPS/PT positivity.

Statistical significance was considered for *p* < 0.05. All analyses were performed using SPSS version 26.0 (IBM, Armonk, NY, USA).

## Results

The demographic and clinical characteristics of the included patients are described in Table 1.

**Table 1:** Clinical and demographic characteristics.

	**SLE aPS/PT positive**		**SLE aPS/PT negative**	
	**(*N* = 18)**	**%**	**(*N* = 34)**	**%**
**Age**				
Years (mean ±*SD*)	47.5 ± 11.5		44.8 ± 13.5	
**Female**				
(*N*; %)	14	77.8	28	82.4
**aPS/PT IgG**				
Positive (*N*; %)	11	61.1		
Titer (mean ±*SD*; median [range])	99.8 ± 77.8; 121 [12–229]			
**aPS/PT IgM**				
Positive (*N*; %)	16	88.9		
Titer (mean ±*SD*; median [range])	126.2 ± 150.8; 131 [8–518]			
**Lupus anti-coagulant**				
Positive (*N*; %)	5	27.8	12	35.3
**SLE manifestation**				
Skin (*N*; %)	6	33.3	8	23.5
Joints (*N*; %)	17	94.4	28	82.4
Hematological (*N*; %)	3	16.7	6	17.6
Lupus Nephritis^*^ (*N*; %)	8	44.4	11	32.4
Sierositis (*N*; %)	4	22.2	7	20.6
Follow-up^**^				
Years (mean ±*SD*; median [range])	3.7 ± 1.2		3.9 ± 1.1	
**SLE disease duration**				
Years (mean ±*SD*; median [range])	15.7 ± 8.1		19.7 ± 7.2	
Therapy^**^				
Hydroxychloroquine (*N*; %)	17	94.4	33	97.1
Prednisone <7.5 mg/die^***^ (*N*; %)	14	77.8	22	64.7
Cyclophosphamide (*N*; %)	2	11.1	2	5.9
Mycophenolate (*N*; %)	3	16.7	3	8.8
Azathioprine (*N*; %)	6	33.3	10	29.4
Methotrexate (*N*; %)	3	16.7	5	14.7
Rituximab (*N*; %)	5	27.8	10	29.4
Belimumab (*N*; %)	3	16.7	5	14.7
**Thrombotic risk factors**				
Arterial hypertension (*N*; %)	7	38.9	10	29.4
Hyperlipidemia (*N*; %)	2	11.1	5	14.7
Smoking habit (*N*; %)	3	16.7	5	14.7
Diabetes (*N*; %)	0	0.0	0	0.0
Hormone replacement therapy (*N*; %)	0	0.0	0	0.0
Inherited thrombophilia (*N*; %)	0	0.0	0	0.0

* *Biopsy-proven*.

** *After aPS/PT testing*.

*** *For at least 80% of the observation time*.

*No statistical difference was observed between the two groups*.

*S.D., standard deviation; N., number; aPS/PT, anti-phosphatidylserine/prothrombin; Ig, immunoglobulins; SLE, Systemic Lupus Erythematosus*.

This study included a total of 52 patients with SLE [42 (80.8%) females]. Of those, 18 patients (34.6%) were found to be positive for aPS/PT (IgG and/or IgM).

During a mean follow-up of more than 3.5 years (3.9 ± 1.1 years), three patients developed TEs (1.3% per year). The overall cumulative incidence of TEs was 5.8% after 2 years, rising to 16.7% when focusing only on aPS/PT positive SLE patients. Details on the three patients who developed a TE are shown in Table 2. All the TEs events (two cerebrovascular events and one thrombotic kidney microangiopathy) occurred in the aPS/PT positive group. Two patients, one aPS/PT positive and one aPS/PT negative, experienced superficial thrombophlebitis, not included among endpoints. No patient died and no pregnancy was recorded during the follow-up. To confirm the absence of solid aβ2GPI dependent aPL positive test, all patients were tested for aβ2GPI-D1, and they all had negative results.

**Table 2:** Main clinical characteristics of the three patients who developed thromboembolic events.

**Patient**	**Previous clinical SLE manifestation**	**TEs**	**aPL profile**	**Treatment at the time of TE**	**SLEDAI-2K time of last appointment before TE**
#1, F, 49 yrs	Joint, malar rash, oral afthosis	Extended ischemic stroke involving cortico-subcortical occipital area at the level of the left hippocampal gyrus and the internal capsular area	Inconstant LA, aPS/PT IgG, IgM	HCQ, PDN 5 mg	0 (last appointment 2 months before)
#2, F, 36 yrs	Oral aphthosis, photosentivity, malar rash, pleurlal-pericarditis, LN (class IV+V), join, skin	Ischemic stroke (middle cerebral artery territory)	LA, aPS/PT IgG, IgM	HCQ, PDN 5 mg	2 (low C3, last appointment 3 months before)
#3, F, 38 yrs	Photosentivity, malar rash, sub-acute skin rash, pericarditis, joints	Renal TMA	LA, aPS/PT IgG, IgM	HCQ, PDN 5 mg, MTX	4 (low C3, positive anti-dsDNA, last appointment 2 months before)

*TMA, thrombotic microangiopathy; aPL, antiphospholipid antibodies; LA, lupus anti-coagulant; aPS/PT, anti-phosphatidylserine/prothrombin; SLE, systemic lupus erythematosus; TE, thrombotic event; F, Female; SLEDAI, systemic lupus erythematosus disease index; Ig, immunoglobulins; HCQ, hydroxychloroquine; PDN, prednisone; MTX, Methotrexate; anti-dsDNA, anti-double strand DNA*.

No statistically significant difference was observed between patients with TEs compared to those without when dividing for demographic variables (age, sex), SLE features (active SLE assessed by SLEDAI-2K > 6), and arterial and venous risk factors (presence of any additional risk factor, to include smoking, arterial hypertension, hyperlipidemia, diabetes, immobilization).

Although we observed a trend for aPS/PT in conferring an increased risk for TEs (crude HR 12.9, 95% CI 0.7–236.7; *p* = 0.08), the results failed to reach statistical significance. When focusing on IgG aPS/PT, we found that patients who tested positive were at a significantly higher risk for TEs (crude HR 19.6, 95% CI 1.1–357.6; *p* = 0.04) compared to aPS/PT negative patients. When taking the whole follow-up period into account by log-rang analysis, patients with aPS/PT presented with a shorter time free from events. Patients with TEs had a higher GAPSS when compared to those without [6 ± 2.6 vs. 2 ± 5.4; *p* = 0.09]; however, this failed to reach statistical significance.

## Discussion

In clinical practice, assessing thrombotic risk is challenging for patients who tested negative for aβ2GPI-dependent aPL. The clinical course in persons with high-risk aPL profiles (triple positive patients) has been well-described (8); the role of extra-criteria aPL, with or without concomitant positive LA, and their clinical impact on positive subjects has been the subject of debate over the last decades, with heterogeneous conclusions (2).

To address this issue, we prospectively evaluated a cohort of SLE patients followed at our Center. They were homogeneous in terms of strict inclusion criteria and negative for solid assay criteria aPL (aCL and aβ2GPI antibodies), regardless of their LA status. Besides, aPS/PT positivity tests were confirmed 12 weeks apart. Our results show a relevant incidence of TEs during the follow-up period, with the incidence of TEs at 5.8% after 2 years. The annualized incidence of TEs in SLE patients negative for aβ2GPI-dependent aPL testing was 1.3%. When focusing on patients who tested positive for aPS/PT, the incidence of TEs rises to 16%, with an annualized incidence of 2.8%, with aPS/PT IgG isotype strongly associated with an increased thrombotic risk. To our knowledge, this is the first prospective clinical study that addresses the incidence of TEs in patients positive for aPS/PT and negative for aβ2GPI-dependent aPL testing. Interestingly, in our cohort, LA positivity did not seem to confer an additional risk for TEs.

Additionally, in a prospective study (9), Ruffatti et al. reported that arterial hypertension and LA positivity were independent risk factors for thrombosis when investigating the risk factors for a first thrombotic event in aPL antibody carriers, most of whom had an associated autoimmune disease. While it is clear that LA positivity is associated with TEs (10), managing patients with isolated LA still requires some considerations (11). Investigating the comprehensive aPL profile of patients/carriers should be mandatory, as the isolated positivity for LA has not been unanimously associated with thrombosis (12) or with clinical manifestations of APS (13). Similar findings were observed in the Leiden thrombophilia case-control study (14), which showed that LA positivity in the absence of aβ2GPI or anti-prothrombin antibodies was not associated with an increased risk for deep vein thrombosis. The association of aPS/PT with thrombosis, especially venous thrombosis, was stronger in the LA positive patients than in LA negative subjects. We observed that aPS/PT was independently associated with thrombosis and pregnancy loss after multivariate analysis (15).

This study has some limitations, including the number of observed events in the relatively short follow-up and sample size (albeit in line with the low prevalence of APS, especially when focusing on subgroups of patients with specific aPL profiles). For instance, while no statistical significance was found when looking at the higher levels of GAPSS in patients with TEs, this probably was due to sample size. Furthermore, patients included in the study had SLE in association with APS, which could have influenced the outcome. These observations require further validation in cohorts of patients without concomitant SLE. Investigation of any change in aPS/PT titres after the second confirmation was outside the scope of this study. These aspects were, however, counterbalanced by the use of strict inclusion criteria (including aPS/PT positivity confirmation at least 12 weeks) and the prospective nature of the study. Finally, since consecutive patients with SLE who met the inclusion criteria were prospectively enrolled in the study, some degree of variability in follow-up length was present. Future studies with a larger sample population, homogenous follow-up duration, and accordingly designed statistical analysis plans are warranted to obtain definite conclusions.

This study observed a rate of TEs of 1.3% each year only in aPS/PT positive patients. This prospective data is validated by previous retrospective studies (3, 4) and suggests that aPS/PT might confer an increased risk for the development of TEs in SLE patients. Future research should investigate whether SLE patients with aPS/PT could benefit from tailored primary thrombo-prophylaxis strategies to include anti-platelet agents. Large clinical trials are needed in the future to test this hypothesis.

## Data Availability Statement

The raw data supporting the conclusions of this article will be made available by the authors, without undue reservation.

## Ethics Statement

The studies involving human participants were reviewed and approved by Local legislation of Rare Diseases in Piedmont (Northwest Italy) (protocol. n. 1577/UC/SAN 11.10.2005). The patients/participants provided their written informed consent to participate in this study.

## Author Contributions

SS and MR designed the study and made the figures. IC, ER, AB, SF, AV, and DRos carried out clinical follow-up, collected data, reviewed the manuscript, and interpreted data. SS, DRoc, and MR analyzed the data. DRoc and SS drafted and revised the paper. All authors approved the final version of the manuscript.

## Conflict of Interest

The authors declare that the research was conducted in the absence of any commercial or financial relationships that could be construed as a potential conflict of interest.
